# Relationship Between Hardiness and Social Anxiety in Chinese Impoverished College Students During the COVID-19 Pandemic: Moderation by Perceived Social Support and Gender

**DOI:** 10.3389/fpsyg.2022.926863

**Published:** 2022-07-20

**Authors:** Xiaoshuang Cheng, Jingxuan Liu, Jun Li, Ziao Hu

**Affiliations:** ^1^ Media and Communication College, Yunnan Normal University, Kunming, China; ^2^ Hainan Vocational University of Science and Technology, Haikou, China

**Keywords:** hardiness, social anxiety, perceived social support, gender, Chinese impoverished college students

## Abstract

During the COVID-19 epidemic, quarantine and financial disadvantages might exacerbate social anxiety among impoverished college students. Based on the hardiness model and the social support buffering model, the present study proposed and verified a dual moderation model to investigate the effects of hardiness on social anxiety and the moderating roles of gender and perceived social support. The hardiness scale, the perceived social support scale, and the social anxiety subscale of the self-consciousness scale were administered to 673 Chinese college students aged 18 to 23 years who were recognized as impoverished by the Chinese authorities and provided with funding. The results revealed that (1) hardiness had a significant negative effect on social anxiety, (2) perceived social support moderated the effect of hardiness on social anxiety, and (3) gender moderated the effect of hardiness on social anxiety. The dual moderated model proposed in the study provides practical implications for helping impoverished college students cope with social anxiety during the COVID-19 pandemic.

## Introduction

The alleviation of poverty and its adverse effects is a concern worldwide (Rubenstein, 2013). Additionally, the mental health problems associated with poverty need to be considered (Burns, 2015). With the implication of targeted poverty alleviation in China, Chinese impoverished college students (CICSs) have received material support. The CICSs refer to students with insufficient financial capacity to meet the basic expenses of study and life in school (Ministry of Education of the PRC and Ministry of Finance of the PRC, 2007). Once universities and local authorities officially recognize these students as “impoverished or CICSs” based on government documents, they are eligible to receive funds from the Chinese government and other sources (Ministry of Education of the PRC et al., 2018). In 2020, subsidies were provided to 36,782,200 CICSs nationwide by the national and provincial governments, universities, and foundations to help them better endure the practical difficulties of the pandemic (China National Center for Student Financial Aid, 2021). Although CICSs receive material support, they are still prone to psychological problems (Tan and Wu, 2017) and face psychological poverty (Li and Xu, 2017). Several studies have revealed that CICSs have worse mental health than the average college student (Zhang, 2000; Hu, 2010; Wang et al., 2015; Cheng et al., 2021). They tend to isolate themselves due to low self-esteem as well as high anxiety and sensitivity (Liu and Tian, 2011), avoid social activities (Luo et al., 2009), and experience social anxiety (Zeng et al., 2017). During the COVID-19 pandemic, Chinese college students experienced different degrees of anxiety (Jin et al., 2021; Pan et al., 2021), and anxiety symptoms were more pronounced in CICSs than in regular students (Liu et al., 2021).

In order to ensure the quality of learning, the Chinese education administration requires college students in low-risk regions to study on campus rather than online. Colleges and universities implement control measures such as quarantining students on campus during the academic semester to prevent the risk of infection. According to the self-presentation theory of social anxiety (Schlenker and Leary, 1982), socially anxious people exhibit certain emotions and behaviors in social encounters because of concerns about judgment from others. Under the order of quarantine on campus, CICSs are inevitably exposed to social situations in dormitories (usually 4–6 students share one room). Besides, researchers have also noted high family socioeconomic status was a mitigating factor for severe social anxiety during COVID-19 lockdowns (Itani et al., 2021). Hence, quarantine on campus and financial disadvantages might exacerbate social anxiety among CICSs under the COVID-19 epidemic background. Social anxiety adversely influences college students (Damer et al., 2010). It leads to negative externalizing behaviors, such as verbal and physical aggression, anger, hostility (Li and Ji, 2015), study difficulties and dropouts in severe cases (Davidson et al., 1993; Himle et al., 2020). It also leads to internalizing problems, including depressive symptoms or depression (Rapee et al., 2019), loneliness or social isolation (Stoeckli, 2010), and fear of positive evaluation (Weeks et al., 2008). Therefore, the social anxiety in CICSs during the pandemic is of significant concern.

### Hardiness and Social Anxiety

The concept of hardiness was first used in agronomy, referring to the ability of crops to resist adverse conditions (Low, 1996). Kobasa introduced hardiness into psychology and defined it as a set of personality traits that help people manage their attitudes, beliefs, and behaviors in stressful situations (Kobasa, 1979; Maddi and Kobasa, 1984). Previous researchers regarded cognitive hardiness (Beasley et al., 2003) or personal hardiness (Maddi, 2013) as the basis of resilience. As was established by the hardiness model (Maddi, 2002; Kinder, 2005), hardiness can strengthen resilience by mitigating stress-triggered adverse health effects (Kobasa, 1979; Kobasa et al., 1981; Kobasa and Puccetti, 1983; Maddi and Kobasa, 1984; Bigbee, 1985; Wiebe, 1991; Maddi and Khoshaba, 1994; Bartone, 1999; Eschleman et al., 2010). Several studies have discussed the relationship between hardiness and anxiety. For instance, Hanton et al. (2013) discovered lower anxiety levels in the high-hardiness college athlete group. Dursun et al. (2022) reported that hardiness scores were remarkably lower in patients with generalized anxiety disorder. Kowalski and Schermer (2019) confirmed that hardiness was negatively associated with anxiety among the college student population.

As a crucial subtype of anxiety, social anxiety often manifests in social situations that even individuals in a nonclinical population can experience (Leary, 1990; Purdon et al., 2001). In the present study, social anxiety is defined as the emotional response (e.g., nervousness, fear, shyness) and behavioral response (e.g., avoidance of social encounters) that can occur when a person faces a social situation (Watson and Friend, 1969; Leitenberg, 1990). According to the self-presentation theory of social anxiety (Schlenker and Leary, 1982), socially anxious people often have a strong desire to make a favorable impression on others but are deeply concerned about the negative evaluation or criticism. Among the CICSs discussed in the present study, social anxiety is one of the most common social disorders (Zeng et al., 2017). Studies have reported that resilience can predict social anxiety (Ko and Chang, 2019; Yu et al., 2019). As the pathway to resilience (Maddi, 2013), hardiness can also predict social anxiety. For instance, Neissi et al. (2005) discovered that hardiness was negatively related to social anxiety and argued that hardiness was one of the best predictors of social anxiety by surveying 200 first-year female high-school students. Therefore, the current study explored the relationship between hardiness and social anxiety in the CICSs population. Given the above discussion, hypothesis 1 (H1) was proposed: *Hardiness significantly negatively affects social anxiety in CICSs.*

### Moderating Role of Perceived Social Support

Perceived social support in the present study refers to an individual’s subjective perception of social support and psychological resources such as care, attention, and respect from others (Barrera, 1986; Zimet et al., 1988; Malecki and Demary, 2002). The buffering model of social support (Cohen and Wills, 1985) reveals that perceived social support can help individuals cope with stressful situations. The buffering effect is independent of the amount of support an individual receives but dependent upon satisfaction with the support available (Sarason et al., 1983). The perception of being supported by others is often enough to help an individual cope with a problematic situation (Thoits, 1995; Taylor et al., 2004). Research has noted hardiness and social support can protect against stress (Pengilly and Dowd, 2000) and discovered a positive relationship between hardiness and social support (Maddi and Kobasa, 1984). Perceived social support consists of three sources: support from significant others, family, and friend (Zimet et al., 1988). Studies have highlighted that poor relationships with parents (Yu et al., 2019), low support from teachers (Weymouth and Buehler, 2018), and not-close peer friendships (Langston and Cantor, 1989; La Greca and Lopez, 1998) can associate with social anxiety. Although supportive relationships with friends, mothers, and fathers each play their own role in protecting against social anxiety (Van Zalk and Van Zalk, 2015), the cumulative support of three sources is also associated with decreased social anxiety (Cavanaugh and Buehler, 2016). Therefore, perceived social support may weaken the social anxiety of CICSs (Cheng et al., 2021).

However, the relationship between social support and social anxiety is complicated. As Calsyn et al. (2005) demonstrated, the social causation hypothesis implies that social support creates social anxiety; in contrast, the social selection hypothesis postulates that social anxiety causes social support. Additionally, the reciprocal theory suggests a mutually causal relationship between social support and social anxiety (Calsyn et al., 2005). Thus, the role of social support in various relationships regarding social anxiety may be different. While the study illustrated the mediation effects of perceived care from friends on the correlation between making friends and social anxiety (Van Zalk and Van Zalk, 2015), researchers reported the moderating role of perceived social support in the association between social anxiety and mobile phone addiction (Zhou et al., 2021). Ren and Li (2020) confirmed that perceived social support moderated the relation between physical activity and social anxiety among left-behind children in rural China with similar low socioeconomic status and poverty problems as CICSs (Murphy, 2022). Therefore, we suggested that perceived social support might also moderate the association between hardiness and social anxiety in CICSs. Hypothesis 2 (H2) was proposed: *Perceived social support has a moderating effect on the relationship between hardiness and social anxiety in CICSs*.

### Moderating Role of Gender

We included gender in the present study because many studies have reported gender differences in both hardiness and social anxiety. Gender is a complex social construct, and gender roles might partially explain the reported differences (Turk et al., 1998; Weinstock, 1999; Moscovitch et al., 2005). Regarding gender differences in hardiness, a study of survivors from the Sinabung eruption disaster reported that female survivors had higher hardiness levels than male survivors (Muda et al., 2016). A study investigating the relationship between gender traits and hardiness among general Chinese college students revealed that masculinity was strongly associated with hardiness (Chen, 2015). In terms of gender differences in social anxiety, studies have noted that women are more likely to suffer from social anxiety than men (Turk et al., 1998; Asher et al., 2017; Asher and Aderka, 2018). In a sociodemographic profile survey of the Canadian population, female respondents with social anxiety experienced more distress than male respondents (MacKenzie and Fowler, 2013). In an adolescent child population, gender differences were significant in the relationship between attentional bias to threat-relevant information and social anxiety (Zhao et al., 2014). Gender also moderated the relationship between peer attachment and social anxiety (Lu et al., 2015). Although rarely studies have examined gender differences in social anxiety among CICSs, Qiu et al. (2011) identified gender differences in state anxiety levels among CICSs. In light of this discussion, hypothesis 3 (H3) was proposed: *Gender plays a moderating role in the relationship between hardiness and social anxiety in CICSs*.

**Figure 1:**
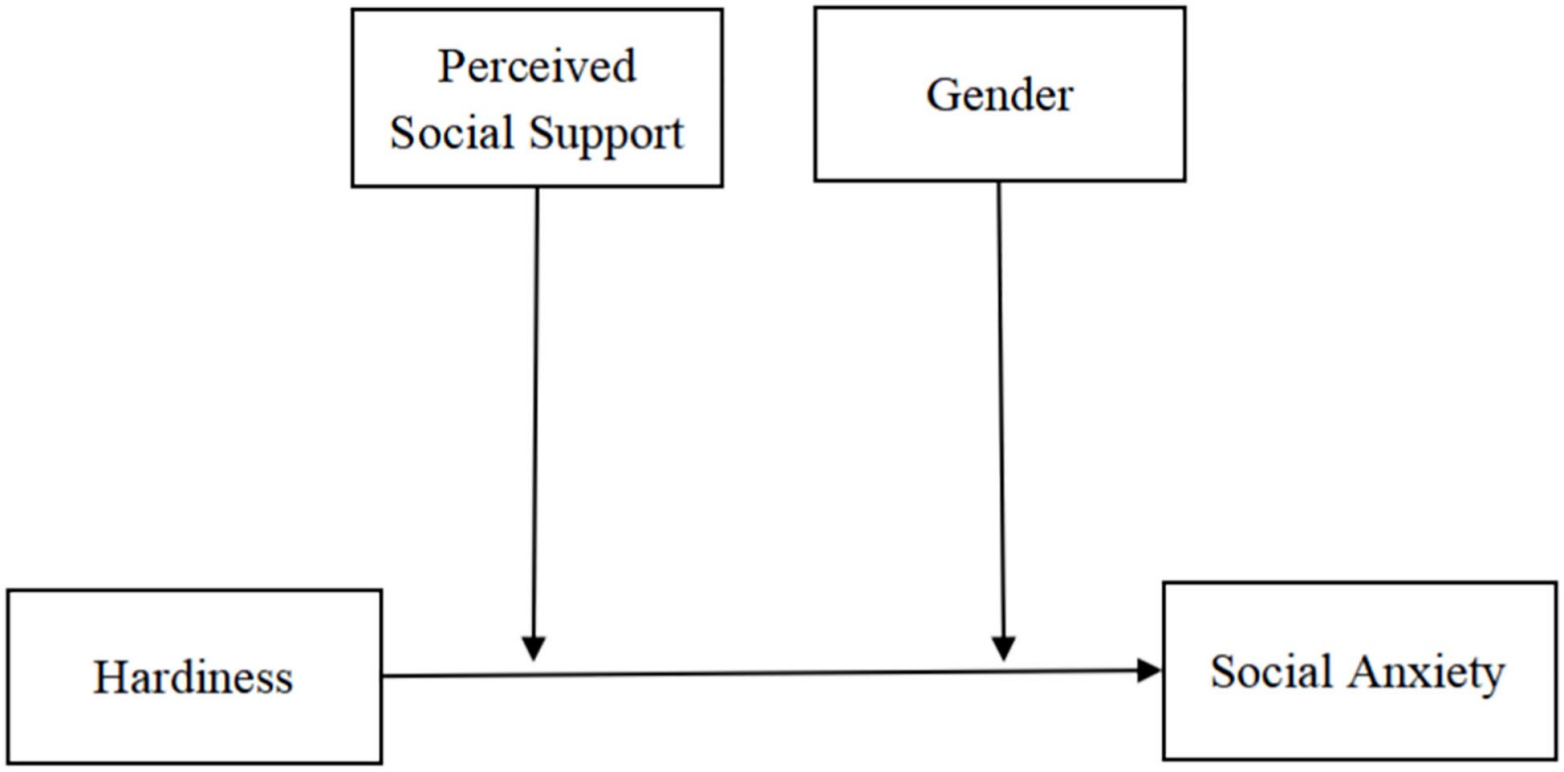
Hypothetical model.

In summary, past studies have separately examined hardiness (e.g., Dursun et al., 2022) and social anxiety (e.g., Himle et al., 2020), and established that hardiness, along with social support, protects against stress-related illnesses (Maddi, 2002). However, to our knowledge, the literature discussing the relationship between hardiness, social support, and social anxiety is limited. Another vital gap lies in the little research focused on impoverished populations. Most literature on hardiness and resilience has focused on business executives and employees (e.g., Maddi and Kobasa, 1984; Chia and Chu, 2017), medical staff (e.g., Hurst and Koplin-Baucum, 2005), athletes (e.g., Nezhad and Besharat, 2010), military personnel (e.g., Bue et al., 2018), patients (e.g., Taheri et al., 2014), teachers (e.g., Chan, 2003), adolescents (e.g., Malkin et al., 2019), and general college students (e.g., Maddi et al., 2009). The research on social anxiety in impoverished populations is also limited (Himle et al., 2020). Therefore, the current study recruited CICSs to explore the relationship between hardiness and social anxiety based on the hardiness model (Maddi, 2002; Kinder, 2005) and the buffer models of social support (Cohen and Wills, 1985). We considered perceived social support and gender to be moderating variables. Figure 1 illustrates the overall hypothetical model.

## Materials and Methods

### Participants

Yunnan province is an impoverished region under the Chinese targeted poverty alleviation policy scheme. It is affected by COVID-19 cases imported from neighboring countries due to its location on the southwest border of China. The data were collected from September 19 to 23 in 2021, when the sampling college was under control measures of closed-off. The purposive sampling method was employed to ensure the participants were: (1) quarantined on campus and (2) officially recognized as “CICSs” before the present study. The college counselors (responsible for psychological counseling for CICSs) assisted the investigators in recruiting participants and distributing online questionnaires through the online chatting rooms of WeChat (a popular Chinese social medium). The college counselors were trained about the study purpose and procedures to control information bias.

Following the Declaration of Helsinki (Goodyear et al., 2007), the current study was conducted with voluntary cooperation considering participants’ privacy and wishes. All participants gave their informed consent for inclusion before participating in the study. They were informed that (1) the study’s purpose, (2) their data would remain confidential, (3) the data would be used for the quantitative statistics in the study and no risks associated, and (4) the study was not compulsive and they could quit the online questionnaire at any time. The online questionnaires were answered and submitted voluntarily and anonymously by participants. Finally, 684 questionnaires were distributed, 11 invalid questionnaires were excluded, and 673 valid questionnaires remained for a return rate of 98%. Among the participants, 135 (20.1%) were male and 538 (79.9%) were female, and the age range was 18–23 years.

### Measures

The hardiness scale, the perceived social support scale, and the social anxiety subscale of the self-consciousness scale were used as measurement instruments. All scales have been validated in prior research with excellent reliability and validity in general college student samples. We performed factor analysis to ensure the fit of the instruments to the test samples due to the specificity of participants in this study.

#### Hardiness Scale

A hardiness scale developed by Lu and Liang (2008) for use with Chinese college students was employed in the present study. The scale comprises four dimensions: control (e.g., “when I encounter difficulties, I always try to find solutions”), challenge (e.g., “I prefer to do work that is full of challenges and often changes”), input (e.g., “I always put much passion into my work”), and resilience (e.g., “I can keep doing difficult tasks as long as it is meaningful”). The scale comprises 27 questions answered using a 5-point Likert scale ranging from 1 (totally inconsistent) to 5 (totally consistent), with higher scores indicating higher levels of hardiness. The Cronbach’s α for the scale in the present study was 0.962, greater than 0.7, indicating favorable reliability (Nunnally and Bernstein, 1994). The results of confirmatory factor analysis (CFA) are presented in Table 1, and the standardized factor loadings (SFLs) were in the range of 0.667–0.836, greater than 0.5, indicating favorable validity (Hair et al., 1992). The composite reliability (CR) values were in the range of 0.896–0.915, greater than 0.6, and the average variance extracted (AVE) values were in the range of 0.558–0.643, greater than 0.5, indicating favorable convergent validity (Fornell and Larcker, 1981). The model fit indices were as follows: χ^2^/*df* = 3.522, root mean residual (RMR) = 0.030, root mean square error of approximation (RMSEA) = 0.061, comparative fit index (CFI) = 0.935, goodness of fit index (GFI) = 0.877, normed fit index (NFI) = 0.912, Tucker-Lewis index (TLI) = 0.929, and parsimonious normed fit index (PNFI) = 0.826, indicating favorable fit (McDonald and Ho, 2002; Hsiao et al., 2016).

**Table 1:** CFA of the hardiness scale.

Dimension	Item	SFL	CR	AVE
Control	1. When I encounter difficulties, I always try to find solutions.	0.766	0.910	0.558
	2. When facing an unfavorable situation, I try to turn the situation around.	0.788		
	3. I can keep my spirits up even when things are not going well.	0.758		
	4. Whenever there is a problem, I try to find its root cause.	0.762		
	5. When someone is angry with me, I try to calm them down.	0.667		
	6. No matter how complicated a problem is, I can always clear my mind quickly.	0.755		
	7. I often regard the difficulties I encounter in life as a challenge rather than a threat.	0.761		
	8. I remain calm in the face of criticism from others.	0.710		
Challenge	1. The changes in my life and work often excite me.	0.732	0.903	0.573
	2. I like to try new and exciting things.	0.724		
	3. I prefer to do work that is full of challenges and often changes.	0.836		
	4. I prefer to be responsible for important work.	0.757		
	5. Breaking the rules inspires me to learn.	0.785		
	6. I am willing to give up a stable life for the chance to face big challenges.	0.721		
	7. Embracing new scenarios in my life is important to me.	0.735		
Input	1. Work and study are fun.	0.788	0.896	0.589
	2. I look forward to working or studying almost every day.	0.743		
	3. I get excited and am positive about working hard.	0.773		
	4. The busy pace of life makes me feel fulfilled.	0.735		
	5. I always put much passion into my work.	0.830		
	6. I put effort into even the simplest things.	0.730		
Resilience	1. I can always achieve my goals through my own efforts.	0.784	0.915	0.643
	2. I can keep doing difficult tasks as long as they are meaningful.	0.829		
	3. I am not afraid of facing difficulties in what I decide to do.	0.819		
	4. I do not easily give up my ideals and goals.	0.813		
	5. If I work hard, any difficulty can be overcome.	0.761		
	6. If I set a goal, I will not give up even if I encounter obstacles.	0.802		

CFA, confirmatory factor analysis; SFL, standardized factor loading; CR, composite reliability; and AVE, average variance extracted.

#### Perceived Social Support Scale

The current study used the perceived social support scale developed by Zimet et al. (1988). The scale contains three dimensions: support from significant others (e.g., “There is a special person who is around when I am in need”), family support (e.g., “I get the emotional help and support I need from my family”), and friend support (e.g., “I can count on my friends when things go wrong”). It contains 12 questions that are scored using a 7-point Likert scale ranging from 1 (totally inconsistent) to 7 (totally consistent). Higher scores indicate higher levels of perceived social support. The Cronbach’s α of the scale in the study was 0.943, greater than 0.7, indicating favorable reliability. Table 2 displays the results of CFA; the SFLs were 0.822–0.884, greater than 0.5, indicating favorable validity. The CR values were 0.909–0.923, greater than 0.6. The AVE values were in the range of 0.715–0.751, greater than 0.5, indicating favorable convergent validity. The model fit indices were: χ^2^/*df* = 4.771, RMR = 0.026, RMSEA = 0.075, CFI = 0.972, GFI = 0.941, NFI = 0.964, TLI = 0.963, and PNFI = 0.745, demonstrating that the scale exhibited favorable fit.

**Table 2:** CFA of the perceived social support scale.

Dimension	Item	SFL	CR	AVE
Significant others	1. There is a special person who is around when I am in need.	0.826	0.909	0.715
	2. There is a special person with whom I can share my joys and sorrows.	0.834		
	3. I have a special person who is a real source of comfort to me.	0.836		
	4. There is a special person in my life who cares about my feelings.	0.884		
Family	1. My family really tries to help me.	0.863	0.914	0.726
	2. I get the emotional help and support I need from my family.	0.869		
	3. I can talk about my problems with my family.	0.822		
	4. My family is willing to help me make decisions.	0.853		
Friends	1. My friends really try to help me.	0.869	0.923	0.751
	2. I can count on my friends when things go wrong.	0.860		
	3. I have friends with whom I can share my joys and sorrows.	0.871		
	4. I can talk about my problems with my friends.	0.865		

CFA, confirmatory factor analysis; SFL, standardized factor loading; CR, composite reliability; and AVE, average variance extracted.

#### Social Anxiety Scale

The present study used the social anxiety subscale of the self-consciousness scale developed by Fenigstein et al. (1975). The scale contains six questions (e.g., “It takes me time to overcome my shyness in new situations”), which are answered using a 5-point Likert scale ranging from 1 (totally inconsistent) to 5 (totally consistent), with higher scores indicating higher levels of social anxiety. The fourth question in the scale was reversed, and the data were analyzed using reverse scoring. The Cronbach’s *α* of the total scale in the study was 0.933, greater than 0.7, indicating favorable reliability. The results of the CFA are presented in Table 3. The SFLs were in the range of 0.737–0.892, greater than 0.5, indicating that the scale had favorable validity. The CR values were 0.933, greater than 0.6. The AVE value was 0.699, greater than 0.5, indicating that the scale had favorable convergent validity. Because this scale is unidimensional, the multifactor oblique intersection model was used to test the overall measurement model fit indicators of this scale and the other two scales, as presented in Table 4. The three scales used in the current study have favorable fit.

**Table 3:** CFA of the social anxiety scale.

Dimension	Item	SFL	CR	AVE
Social anxiety scale	1. It takes me time to overcome my shyness in new situations.	0.737	0.933	0.699
	2. I have trouble working when someone is watching me.	0.799		
	3. I get embarrassed very easily.	0.886		
	4. I do not find it hard to talk to strangers.	0.836		
	5. I feel anxious when I speak in front of a group.	0.892		
	6. Large groups make me nervous.	0.857		

CFA, confirmatory factor analysis; SFL, standardized factor loading; CR, composite reliability; and AVE, average variance extracted.

**Table 4:** Model fit indices of the measurement model.

Standard	χ^2^/*df* < 5	RMR < 0.08	RMSEA < 0.08	CFI > 0.9	GFI > 0.85	NFI > 0.9	TLI > 0.9	PNFI > 0.5	HOELTER.05 > 200
Results	2.624	0.031	0.049	0.936	0.852	0.900	0.930	0.834	277

#### Common Method Variance (CMV) Test

Harman’s one-factor test was used to assess CMV. Unrotated factor analysis revealed that the Kaiser–Meyer–Olkin value was 0.960 (>0.8), and the Bartlett test of sphericity reached significance (*p* < 0.001). The analysis yielded six factors, and the explanatory power of the first factor was 38.813%, which did not exceed the critical value of 50% (Podsakoff et al., 2003), indicating that CMV was not significant in the present study.

#### Statistical Methods

First, descriptive statistical analysis, correlation analysis, scale reliability tests, and CMV tests were conducted using SPSS 21.0, and CFA was performed using AMOS 21.0. Second, the moderating effects of perceived social support were tested using Model 2 of PROCESS, and bootstrap confidence intervals were used to determine whether the two moderating effects in Model 2 were significant (Hayes, 2013).

## Results

### Descriptive Statistics and Correlations for All Variables

The descriptive statistics for the variables are presented in Table 5. The results indicated that the CICSs in the current study had moderate levels of social anxiety and moderate-to-high levels of hardiness and perceived social support during the COVID-19 pandemic. The correlation analysis results indicated that (1) gender was not significantly correlated with any of the other three variables in the current study, indicating the requirement of controlling on gender was not necessary for the regression analysis; (2) hardiness and social anxiety were negatively correlated (correlation coefficient = −0.204; *p* < 0.001), and (3) hardiness and perceived social support were positively correlated (correlation coefficient = 0.569; *p* < 0.001), and (4) perceived social support was negatively correlated with social anxiety (correlation coefficient = −0.088; *p* < 0.05). The absolute values of the correlation coefficients among the three variables were smaller than 0.8, indicating a weak-to-moderate correlation between the variables and no collinearity problem (Cohen et al., 2009).

**Table 5:** Descriptive statistics and correlations for all variables.

Variable	*M*	*SD*	Gender	Hardiness	Social anxiety	Perceived social support
Gender	0.200	0.401	1			
Hardiness	3.851	0.607	0.072	1		
Social anxiety	2.898	1.050	0.042	−0.204^***^	1	
Perceived social support	3.887	0.755	−0.028	0.569^***^	−0.088^*^	1

*n* = 673; Gender was treated as a dummy variable, 1 = male, 0 = female; M, mean; SD, standard deviation.

* *p* < 0.05;

*** *p* < 0.001.

### Differential Analysis for Gender

Since the moderating role of gender is one of the main concerns of this study, descriptive information on hardiness, social anxiety, and perceived social support among males and females are presented in Table 6. The *t*-test of independent samples demonstrated that gender had no significant differences in all three variables. The results confirmed again that the regression analysis in the present study did not require controlling on gender as a background variable.

**Table 6:** Differential analysis for gender in all variables.

Variable	Groups	Hardiness		Perceived social support		Social anxiety	
		M(SD)	*t*	M(SD)	*t*	M(SD)	*t*
Gender	Male	3.938 (0.674)	1.866	3.845 (0.767)	−0.718	2.985 (1.103)	1.076
	Female	3.829 (0.588)				3.897 (0.753)			2.876 (1.037)	

### Moderating Roles of Perceived Social Support and Gender

To illustrate that the current study’s regressive framework and moderation tests were justified, regression analysis hypothesis testing was used to test the linearity, normality, and homogeneity of variance. First, a scatter plot for hardiness and social anxiety demonstrated a negative linear relationship between hardiness and social anxiety in the study. The results indicated that the research data satisfied linearity (Hayes, 2013). Second, the Durbin–Watson value was 2.039 (between 1.5 and 2.5), denoting no autocorrelation. The results indicated that the research data satisfied independence (Tabachnick and Fidell, 2001). Third, the skewness absolute values for the 45 items ranged between 0.025 and 1.007, and the kurtosis absolute values for the 45 items were between 0.010 and 1.928. The results satisfied the standards of the absolute value for skewness <2 and kurtosis <7 (Curran et al., 1996) and indicated that the research data satisfied normality. Finally, the regression standard residual scatter plot was used to test the problem of homogeneity. The scatter plot demonstrated that the residual means were on the same straight line; therefore, the data satisfied the homogeneity of variance assumption (Hayes, 2013).

Model 2 of PROCESS was used to incorporate both perceived social support and gender into one model to test the moderating effects of these variables. The results displayed in Table 7 reveal that hardiness significantly negatively predicted social anxiety (*B* = −0.529; *p* < 0.001). The results were verified using the bias-corrected nonparametric percentile bootstrapping method; the 95% confidence interval (CI) was discovered not to contain 0 (lower limit of CI [LLCI] = −0.722, the upper limit of CI [ULCI] = −0.347). Therefore, H1 was supported.

**Table 7:** Testing the moderating roles of perceived social support and gender.

Variable	Social anxiety		
	B	*t*	95%LLCI 95%ULCI
Hardiness	−0.529	−6.101^***^	(−0.722 -0.347)
Perceived social support	0.049	0.768	(−0.084 0.170)
Hardiness * Perceived social support	−0.189	−3.172^**^	(−0.323 -0.005)
Gender	0.159	1.605	(−0.040 0.350)
Hardiness * Gender	0.347	2.278^*^	(0.004 0.705)
*R^2^*	0.072		
*F*	10.354^***^		

* *p* < 0.05;

** *p* < 0.01;

*** *p* < 0.001.

B are unstandardized coefficients; LLCI, lower limit of confidence interval and ULCI, upper limit of confidence interval.

**Figure 2:**
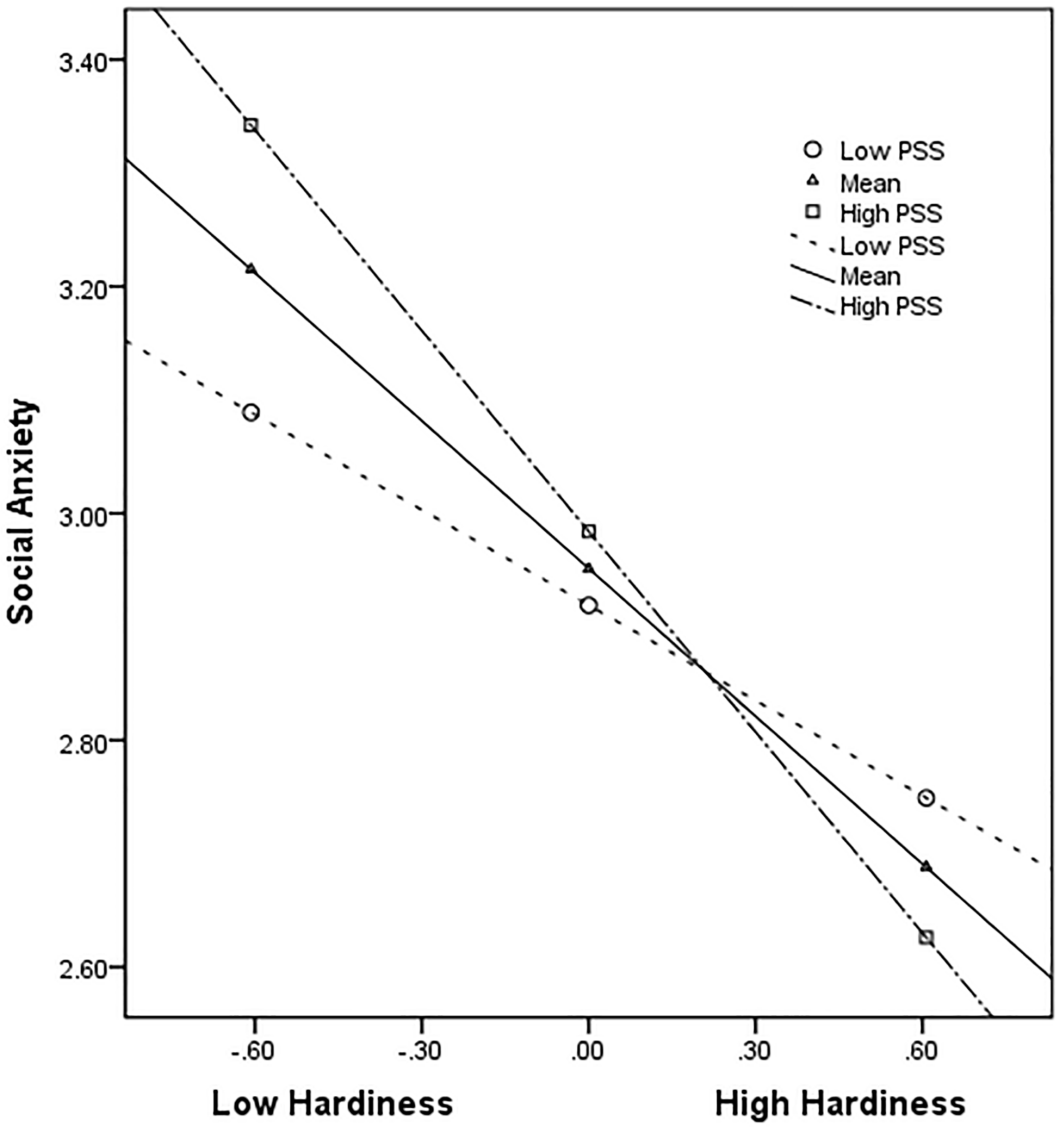
Moderating effect of perceived social support (PSS) on the relationship between hardiness and social anxiety; the moderating effect is plotted for two levels of PSS: high PSS (1 SD above the mean) and low PSS (1 SD below the mean).

The results displayed in Table 7 revealed that the interaction between hardiness and perceived social support exhibited a significant negative predictive effect on social anxiety (*B* = −0.189; *p* < 0.01), which was verified using the bias-corrected nonparametric percentile bootstrap method. The 95% CI did not contain 0 (LLCI = −0.323, ULCI = −0.005), meaning that H2 was supported. Perceived social support moderated the effect of hardiness on social anxiety. The study conducted a simple slope analysis for the relation between hardiness and social anxiety at low and high levels of perceived social support (−1 SD, Mean, +1 SD) to illustrate the interaction effect further. Figure 2 demonstrates that the social anxiety level reduces slightly for CICSs with low perceived social support as the hardiness level improves (simple slope = −0.280; *t* = −3.274; *p* < 0.01). In contrast, for CICSs with a high level of perceived social support, the social anxiety level reduces significantly as the hardiness level improves (simple slope = −0.589; *t* = −6.102; *p* < 0.001). The negative effect of hardiness on social anxiety was stronger for CICSs with high perceived social support than for those with low perceived social support, indicating that perceived social support enhanced the negative effect of hardiness on social anxiety in this study.

**Figure 3:**
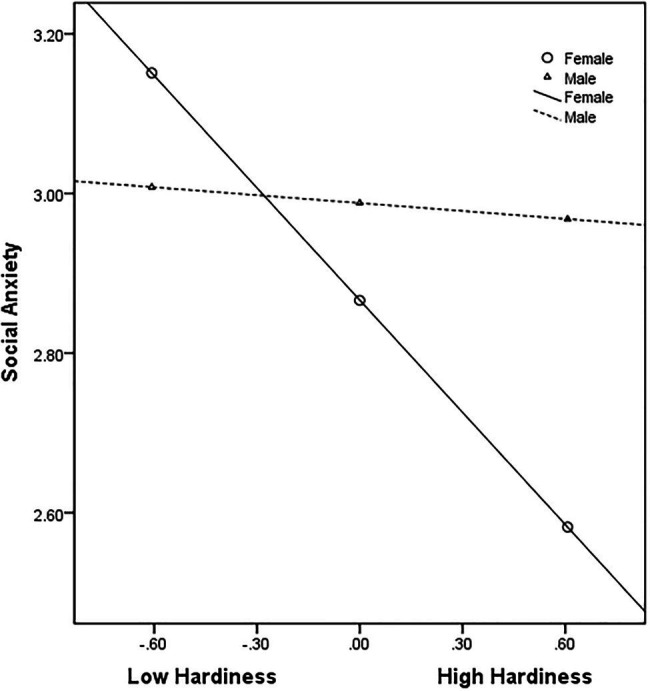
Moderating effect of gender on the relationship between hardiness and social anxiety.

The interaction between hardiness and gender was also a significant predictor of social anxiety (*B* = 0.347; *p* < 0.05). Using the bias-corrected nonparametric percentile bootstrap method, we observed that the 95% CI did not contain 0 (LLCI = 0.004, ULCI = 0.705). The results supported H3, indicating that gender moderated the effect of hardiness on social anxiety. The simple slope analysis was conducted to further explain the moderating effect of gender and the moderating effect was plotted. Figure 3 illustrates that in male CICSs, social anxiety reduces slightly as the hardiness improves (simple slope = −0.032; *t* = −0.247; *p* > 0.05). In contrast, in female CICSs, social anxiety reduces significantly as the hardiness level improves (simple slope = −0.468; *t* = −6.241; *p* < 0.001). The negative effect of hardiness on social anxiety was stronger for female CICSs than for their male peers.

## Discussion

The descriptive statistics results in the current study indicated that social anxiety in CICSs was moderate (*M* = 2.898 out of 5) during the pandemic and deserved focused attention, but little research has been conducted to explore this topic. The present study proposed and verified a dual moderation model to investigate the effect of hardiness on social anxiety in CICSs and tested the moderating roles of perceived social support and gender. The results revealed that hardiness negatively predicts social anxiety in CICSs and that perceived social support and gender moderate this correlation.

### Theoretical Implications

The relationship between hardiness and social anxiety among the impoverished population has rarely been examined in past literature to the best of our knowledge. Our results reveal that hardiness directly affects social anxiety, supporting the hardiness model (Maddi, 2002; Kinder, 2005). Individuals experience social anxiety when stressed with doubts about their ability to make a socially desirable impression on others (Leary, 1995; Schlenker, 2012; Leary and Jongman-Sereno, 2014), and hardiness helps individuals sustain their mental and physical health under stress (Maddi, 2002). Supplementing previous research, which regarded hardiness as a negative predictor of anxiety (Kowalski and Schermer, 2019), our findings demonstrate that hardiness also negatively predicts social anxiety consistent with Neissi et al. (2005). CICSs have received little attention in studies on hardiness or social anxiety. The present study specifically addressed CICSs, and the results confirm that H1 hardiness significantly negatively affects social anxiety in CICSs. This indicates that although economic pressure and quarantined on campus during the COVID-19 pandemic may exacerbate social anxiety in CICSs, hardiness can be an essential protective factor.

Second, our results reveal that the higher the level of perceived social support, the stronger the effect of hardiness on social anxiety. The findings support H2 that perceived social support has a moderating effect on the relationship between hardiness and social anxiety in CICSs, consistent with the social support buffering model (Cohen and Wills, 1985). When social support was measured quantitatively, a direct effect was discovered; by contrast, when social support was constructed qualitatively as perceived social support, the interaction (moderation) effects of the buffering model were reported (Bellman et al., 2003). CICSs may avoid socializing because of the financial and psychological pressure caused by poverty (Luo et al., 2009). Even though China’s poverty alleviation efforts have ensured that CICSs receive various types of material support from the government, CICSs may differ from other students in perceiving social support. The findings suggest that the level of perceived social support compared with actual support, may have cognitive effects on individuals’ social anxiety, which is consistent with previous research (Sarason et al., 1983; Thoits, 1995; Taylor et al., 2004).

Third, our results reveal that the effect of hardiness on the social anxiety of women is greater than it is on men. It supports H3, which states that gender plays a moderating role in the association between hardiness and social anxiety in CICSs. The results might be better understood in the context of gender role theories (Eagly et al., 2000). Scholars have reported gender differences in hardiness personality (e.g., Muda et al., 2016) and social anxiety (e.g., Asher and Aderka, 2018). These gender differences may be related to the gender roles that individuals of both sexes construct through their specific sociocultural upbringing and learning (Carroll and Wolpe, 1996; Eagly and Wood, 1999). Traditionally, masculinity has been linked to personality characteristics such as defending beliefs and being assertive or willing to take risks, whereas femininity has been associated with personality characteristics such as being tender, sensitive, and sympathetic (Bem, 1974). Male gender-role identification mitigates individuals’ perceptions of interpersonal needs, which may lead to underestimating the feelings about social anxiety (Moscovitch et al., 2005). Hence, hardiness may have had a greater effect on the social anxiety of female CICSs. Another likely explanation is that women from Asia were more likely to endorse traditional gender-role attitudes than women in other locations (Robnett and Anderson, 2017). Female CICSs may be more sensitive to interpersonal relationships and social anxiety than male peers, consistent with previous research (Turk et al., 1998; Asher et al., 2017; Asher and Aderka, 2018).

### Practical Implications

The dual moderated model proposed in this study has practical implications for helping impoverished college students cope with social anxiety during the COVID-19 pandemic.

First, hardiness negatively affects social anxiety in CICSs and serves as an essential protective factor. This result has practical implications for psychological health education in colleges and universities. Such institutions should focus on cultivating and improving the hardiness level of impoverished college students during the pandemic. Studies have proved that HardiTraining courses can lead to a tremendous increase in hardiness attitudes and feelings of social support while decreasing anxiety (Maddi et al., 2009). Therefore, colleges may arrange HardiTraining courses to help CICSs confront difficulties and challenges. For instance, the *Situational Reconstruction* activity in the HardiTraining courses can guide trainees to understand the stressful circumstance and be prepared through the imaginary rebuilding of a possible situation (Khoshaba and Maddi, 2001; Maddi et al., 2009). Hence, colleges and universities could organize seminars for CICSs to understand and be prepared for the COVID-19-related difficulties they may face through *Situational Reconstruction* . Additionally, college counselors can guide CICSs to look at the temporary difficulties brought on by the pandemic with an optimistic attitude.

Second, perceived social support moderates the correlation between hardiness and social anxiety in CICSs. Hence, the perceived level of social support of CICSs should be simultaneously improved while providing substantial support. During the pandemic, universities and authorities should guide CICSs to actively recognize the help given by others in their life and studies. College counselors can help them correctly handle interpersonal relationships with teachers, parents, friends, and peers, and obtain emotional support from significant others. The protective effect of hardiness on social anxiety can be strengthened as perceived social support in CICSs is enhanced.

Third, we determined that gender moderates the effect of hardiness on social anxiety in CICSs. Hardiness has a more substantial impact on social anxiety in women than men. Colleges and universities should pay more attention to the gender difference regarding social anxiety issues during the pandemic. Gender-sensitive intervention models can be established to provide targeted psychological support for students of different genders.

## Conclusion

The present study proposed and validated a dual moderation model to explore the mechanism of the effect of hardiness on social anxiety among CICSs during the COVID-19 pandemic. The results revealed that hardiness was significantly and negatively associated with social anxiety, and their relation was moderated by perceived social support and gender. Hardiness plays a protective factor for the social anxiety of a specific group of CICSs. Additionally, the effect of hardiness on social anxiety is stronger for females and individuals with high perceived social support levels. The study also provides some practical suggestions for colleges and universities.

## Limitations and Future Studies

The present study has several limitations. First, the study was a cross-sectional quantitative survey. It reveals the predictive correlations between variables, but it cannot determine their causal relationships. Future studies could employ longitudinal or experimental designs to examine further the causal relationships among the variables. Second, the current study only recruited CICSs as participants. The dual moderation model in the present study could be verified among more diverse samples. Alternatively, a CICS group and a non-CICS group could be compared in future studies. Third, the present study was conducted on samples from Yunnan province, and the generalizability of the findings is limited. The results can be verified in other provinces and countries. Fourth, this study is also limited by its sampling conditions. The participants were from a college where the ratio of male and female students is approximately 1:3. Thus, the gender composition in the samples was unbalanced. Future studies should consider enlarging the geographical scope of sampling or validating our results in different colleges with balanced gender ratios.

## Data Availability Statement

Other data pertaining to this study are available from the corresponding author upon reasonable request.

## Ethics Statement

Ethical review and approval was not mandatory for non-interventional studies (e.g., surveys, questionnaires, social media research) in accordance with the local legislation and institutional requirements. The present study was conducted following the Declaration of Helsinki. All subjects gave their informed consent for inclusion before they participated in the study.

## Author Contributions

XC was the primary author who proposed the research proposal and completed the article for this study. JLiu, JLi, and ZH worked as investigators and writer’s assistants. JLi served as the research advisor. XC, JLiu, JLi, and ZH revised the manuscript was revised collaboratively. All authors contributed to the article and approved the submitted version.

## Funding

This research has been funded by the 2022 annual research project on the students administration of Yunnan Normal University “Publicity Effect and Satisfaction of Financial Aid for Impoverished College Students in Yunnan Normal University” (No. 2022ys40).

## Conflict of Interest

The authors declare that the research was conducted in the absence of any commercial or financial relationships that could be construed as a potential conflict of interest.

## Publisher’s Note

All claims expressed in this article are solely those of the authors and do not necessarily represent those of their affiliated organizations, or those of the publisher, the editors and the reviewers. Any product that may be evaluated in this article, or claim that may be made by its manufacturer, is not guaranteed or endorsed by the publisher.

## References

[ref1] Asher M. Aderka I. M. (2018). Gender differences in social anxiety disorder. J. Clin. Psychol. 74, 1730–1741. doi: 10.1002/jclp.22624 29667715

[ref2] Asher M. Asnaani A. Aderka I. M. (2017). Gender differences in social anxiety disorder: a review. Clin. Psychol. Rev. 56, 1–12. doi: 10.1016/j.cpr.2017.05.004 28578248

[ref3] Barrera M. (1986). Distinctions between social support concepts, measures, and models. Am. J. Community Psychol. 14, 413–445. doi: 10.1007/BF00922627

[ref4] Bartone P. T. (1999). Hardiness protects against war-related stress in army reserve forces. Consult. Psychol. J. 51, 72–82. doi: 10.1037/1061-4087.51.2.72

[ref5] Beasley M. Thompson T. Davidson J. (2003). Resilience in response to life stress: the effects of coping style and cognitive hardiness. Personal. Individ. Differ. 34, 77–95. doi: 10.1016/S0191-8869(02)00027-2

[ref6] Bellman S. Forster N. Still L. Cooper C. L. (2003). Gender differences in the use of social support as a moderator of occupational stress. Stress. Health 19, 45–58. doi: 10.1002/smi.954

[ref7] Bem S. L. (1974). The measurement of psychological androgyny. J. Consult. Clin. Psychol. 42, 155–162. doi: 10.1037/h0036215, PMID: 4823550

[ref8] Bigbee J. L. (1985). Hardiness a new perspective in health promotion. Nurse Pract. 10, 51–56. doi: 10.1097/00006205-198511000-00006, PMID: 4069460

[ref9] Bue S. L. Kintaert S. Taverniers J. Mylle J. Delahaij R. Euwema M. (2018). Hardiness differentiates military trainees on behavioural persistence and physical performance. Int. J. Sport Exerc. Psychol. 16, 354–364. doi: 10.1080/1612197X.2016.1232743

[ref10] Burns J. K. (2015). Poverty, inequality and a political economy of mental health. Epidemiol. Psychiatr. Sci. 24, 107–113. doi: 10.1017/S2045796015000086, PMID: 25746820 PMC6998107

[ref11] Calsyn R. J. Winter J. P. Burger G. K. (2005). The relationship between social anxiety and social support in adolescents: a test of competing causal models. Adolescence 40, 103–113. 15861620

[ref12] Carroll J. L. Wolpe P. R. (1996). Sexuality and Gender in Society. New York: Harper Collins College Publishers.

[ref13] Cavanaugh A. M. Buehler C. (2016). Adolescent loneliness and social anxiety: The role of multiple sources of support. J. Soc. Pers. Relatsh. 33, 149–170. doi: 10.1177/0265407514567837

[ref14] Chan D. W. (2003). Hardiness and its role in the stress-burnout relationship among prospective Chinese teachers in Hong Kong. Teach. Teach. Educ. 19, 381–395. doi: 10.1016/S0742-051X(03)00023-4

[ref15] Chen J. (2015). Correlation between gender role and hardiness of university students. Chin. J. School Health 36, 1839–1841. doi: 10.16835/j.cnki.1000-9817.2015.12.027

[ref16] Cheng J. W. Guo K. D. Gao L. (2021). Relationship between social support and mental health 389 of impoverished vocational college students. Chin. J. Health Psychol. 29, 152–156. doi: 10.13342/j.cnki.cjhp.2021.01.028

[ref17] Chia Y. M. Chu M. J. T. (2017). Presenteeism of hotel employees: interaction effects of empowerment and hardiness. Int. J. Contemp. Hosp. Manag. 29, 2592–2609. doi: 10.1108/IJCHM-02-2016-0107

[ref18] China National Center for Student Financial Aid (2021). Report on the development of financial aid for Chinese students (2020). Available at: http://www.xszz.cee.edu.cn/index.php/shows/70/7264.html (Accessed March 10, 2022).

[ref19] Cohen I. Huang Y. Chen J. Benesty J. (2009). Pearson Correlation Coefficient. Berlin: Springer Berlin Heidelberg.

[ref20] Cohen S. Wills T. A. (1985). Stress, social support, and the buffering hypothesis. Psychol. Bull. 98, 310–357. doi: 10.1037/0033-2909.98.2.310 3901065

[ref21] Curran P. J. West S. G. Finch J. F. (1996). The robustness of test statistics to nonnormality and specification error in confirmatory factor analysis. Psychol. Methods 1, 16–29. doi: 10.1037/1082-989X.1.1.16

[ref22] Damer D. E. Latimer K. M. Porter S. H. (2010). “Build your social confidence”: a social anxiety group for college students. J. Spec. Group Work. 35, 7–22. doi: 10.1080/01933920903463510

[ref23] Davidson J. R. T. Hughes D. L. George L. K. Blazer D. G. (1993). The epidemiology of social phobia: findings from the Duke epidemiological catchment area study. Psychol. Med. 23, 709–718. doi: 10.1017/S0033291700025484, PMID: 8234577

[ref24] Dursun P. Alyagut P. Yilmaz I. (2022). Meaning in life, psychological hardiness and death anxiety: individuals with or without generalized anxiety disorder (GAD). Curr. Psychol. 41, 3299–3317. doi: 10.1007/s12144-021-02695-3, PMID: 35035188 PMC8742667

[ref25] Eagly A. H. Wood W. (1999). The origins of sex differences in human behavior: evolved dispositions versus social roles. Am. Psychol. 54, 408–423. doi: 10.1037/0003-066X.54.6.408

[ref26] Eagly A. H. Wood W. Diekman A. B. (2000). “Social role theory of sex differences and similarities: a current appraisal,” in The Developmental Social Psychology of Gender. eds. Eckes T. Trautner H. M. (Mahwah, NJ: Erlbaum), 123–174.

[ref27] Eschleman K. J. Bowling N. A. Alarcon G. M. (2010). A meta-analytic examination of hardiness. Int. J. Stress. Manag. 17, 277–307. doi: 10.1037/a0020476

[ref28] Fenigstein A. Scheier M. F. Buss A. H. (1975). Public and private self-consciousness: assessment and theory. J. Consult. Clin. Psychol. 43, 522–527. doi: 10.1037/h0076760

[ref29] Fornell C. Larcker D. F. (1981). Evaluating structural equation models with unobservable variables and measurement error. J. Market. Res. 18, 39–50. doi: 10.1177/002224378101800104

[ref30] Goodyear M. D. Krleza-Jeric K. Lemmens T. (2007). The declaration of Helsinki. BMJ 335, 624–625. doi: 10.1136/bmj.39339.610000.BE, PMID: 17901471 PMC1995496

[ref31] Hair J. F. Anderson R. E. Tatham R. L. Black W. C. (1992). Multivariate Data Analysis. Upper Saddle River, NJ: Prentice-Hall.

[ref32] Hanton S. Neil R. Evans L. (2013). Hardiness and anxiety interpretation: an investigation into coping usage and effectiveness. Eur. J. Sport Sci. 13, 96–104. doi: 10.1080/17461391.2011.635810

[ref33] Hayes A. F. (2013). Introduction to Mediation, Moderation, and Conditional Process Analysis: A Regression-Based Approach. New York: Guilford Press.

[ref34] Himle J. A. Weaver A. Levine D. S. Steinberger E. Bybee D. Vlnka S.. (2020). Social anxiety and work: a qualitative investigation in a low-income, minority sample. Soc. Work. Ment. Health 18, 302–330. doi: 10.1080/15332985.2020.1742850 PMC12312657 40746954

[ref35] Hsiao C. Lee Y. H. Chen H. H. (2016). The effects of internal locus of control on entrepreneurship: the mediating mechanisms of social capital and human capital. Int. J. Hum. Resour. Manag. 27, 1158–1172. doi: 10.1080/09585192.2015.1060511

[ref36] Hu C. H. (2010). Multi-comparison of incidence of mental problems between the impoverished and non-impoverished undergraduates. Chin. J. Health Psychol. 18, 87–89. doi: 10.13342/j.cnki.cjhp.2010.01.041

[ref37] Hurst S. Koplin-Baucum S. (2005). A pilot qualitative study relating to hardiness in ICU nurses hardiness in ICU nurses. Dimens. Crit. Care Nurs. 24, 97–100. doi: 10.1097/00003465-200503000-00011, PMID: 15827433

[ref38] Itani M. H. Eltannir E. Tinawi H. Daher D. Eltannir A. Moukarzel A. A. (2021). Severe social anxiety among adolescents during COVID-19 lockdown. J. Patient Exp. 8, 1–10. doi: 10.1177/23743735211038386, PMID: 34568549 PMC8460965

[ref39] Jin Y. L. Chang W. W. Chang X. Zhu L. J. Fang Z. M. Chen Y.. (2021). Analysis of mental health and influencing factors of college students in the online learning period during the outbreak of COVID-19. Chin. J. School Health 42, 574–578. doi: 10.16835/j.cnki.1000-9817.2021.04.022

[ref40] Khoshaba D. M. Maddi S. R. (2001). HardiTraining. Irvine, CA: Hardiness Institute.

[ref41] Kinder R. A. (2005). Psychological hardiness in women with paraplegia. Rehabil. Nurs. 30, 68–72. doi: 10.1002/j.2048-7940.2005.tb00362.x, PMID: 15789699

[ref001] Ko C.-Y. A. Chang Y. S. (2019). Investigating the relationships among resilience, social anxiety, and procrastination in a sample of college students. Psychol. Rep. 122, 231–245. doi: 10.1177/0033294118755111 29375028

[ref42] Kobasa S. C. (1979). Stressful life events, personality, and health: an inquiry into hardiness. J. Pers. Soc. Psychol. 37, 1–11. doi: 10.1037/0022-3514.37.1.1, PMID: 458548

[ref43] Kobasa S. C. Maddi S. R. Courington S. (1981). Personality and constitution as mediators in the stress illness relationship. J. Health Soc. Behav. 22, 368–378. doi: 10.2307/2136678, PMID: 7320474

[ref44] Kobasa S. C. Puccetti M. A. (1983). Personality and social resources in stress resistance. J. Pers. Soc. Psychol. 45, 839–850. doi: 10.1037/0022-3514.45.4.839, PMID: 6631665

[ref45] Kowalski C. M. Schermer J. A. (2019). Hardiness, perseverative cognition, anxiety, and health-related outcomes: a case for and against psychological hardiness. Psychol. Rep. 122, 2096–2118. doi: 10.1177/0033294118800444, PMID: 30253687

[ref46] La Greca A. M. Lopez N. (1998). Social anxiety among adolescents: linkages with peer relations and friendships. J. Abnorm. Child Psychol. 26, 83–94. doi: 10.1023/A:1022684520514, PMID: 9634131

[ref47] Langston C. A. Cantor N. (1989). Social anxiety and social constraint: when making friends is hard. J. Pers. Soc. Psychol. 56, 649–661. doi: 10.1037/0022-3514.56.4.649

[ref48] Leary M. R. (1990). Responses to social exclusion: social anxiety, jealousy, loneliness, depression, and low self-esteem. J. Soc. Clin. Psychol. 9, 221–229. doi: 10.1521/jscp.1990.9.2.221

[ref49] Leary M. R. (1995). Self-Presentation: Impression Management and Interpersonal Behavior. Boulder, CO: Westview Press.

[ref50] Leary M. R. Jongman-Sereno K. P. (2014). “Chapter 20 - social anxiety as an early warning system: a refinement and extension of the self-presentation theory of social anxiety,” in Social Anxiety. 3rd Edn. eds. Hofmann S. G. DiBartolo P. M. (Academic Press), 579–597. doi: 10.1016/B978-0-12-394427-6.00020-0

[ref51] Leitenberg H. (1990). Handbook of Social and Evaluation Anxiety. Boston, MA, US: Springer.

[ref52] Li J. W. Ji Y. (2015). “Research on correlation between social anxiety and aggressive behavior of college students,” in Proceedings Paper of the 2nd International Conference on Education and Education Research (EER 2015). Vol. 9. ed. Zhang Y. (Aalto University), 90–94.

[ref53] Li X. S. Xu X. P. (2017). “Research on the factors and countermeasures of psychological poverty of poor students in colleges and universities,” in Proceedings Paper of the 4th International Conference on Education, Management and Computing Technology (ICEMCT 2017). Vol. 101. ed. Chu X. (Atlantis Press), 1332–1336.

[ref54] Liu H. J. Chen J. He Z. M. (2021). Study on mental health status and influencing factors of university students during COVID-19. Chin. Safety Sci. J. 31, 168–173.

[ref55] Liu F. Tian Z. P. (2011). Psychological assistance for “students of psychological poverty” from impoverished families in colleges and universities. Heilongjiang Res. High. Educ. 207, 137–139.

[ref56] Low J. (1996). The concept of hardiness: a brief but critical commentary. J. Adv. Nurs. 24, 588–590. doi: 10.1046/j.1365-2648.1996.22820.x, PMID: 8876420

[ref57] Lu G. H. Liang B. Y. (2008). Development of hardiness scale. Stud. Psychol. Behav. 2, 103–106+160.

[ref58] Lu A. T. Tian H. P. Yu Y. P. Feng Y. Hong X. X. Yu Z. W. (2015). Peer attachment and social anxiety: gender as a moderator across deaf and hearing adolescents. Soc. Behav. Pers. 43, 231–239. doi: 10.2224/sbp.2015.43.2.231

[ref60] Luo F. S. Shen D. Zhang S. M. (2009). A study on mental health status of impoverished college students and its influencing factors. Chin. J. Clin. Psych. 3, 272–274.

[ref61] MacKenzie M. B. Fowler K. F. (2013). Social anxiety disorder in the Canadian population: exploring gender differences in sociodemographic profile. J. Anxiety Disord. 27, 427–434. doi: 10.1016/j.janxdis.2013.05.006, PMID: 23768484

[ref62] Maddi S. R. (2002). The story of hardiness: twenty years of theorizing, research, and practice. Consult. Psychol. J.: Practice Res. 54, 173–185. doi: 10.1037/1061-4087.54.3.173

[ref63] Maddi S. (2013). “Personal hardiness as the basis for resilience,” in Hardiness. SpringerBriefs in Psychology (Dordrecht: Springer)

[ref64] Maddi S. R. Harvey R. H. Khoshaba D. M. Fazel M. Resurreccion N. (2009). Hardiness training facilitates performance in college. J. Posit. Psychol. 4, 566–577. doi: 10.1080/17439760903157133

[ref65] Maddi S. R. Khoshaba D. M. (1994). Hardiness and mental health. J. Pers. Assess. 63, 265–274. doi: 10.1207/s15327752jpa6302_6 7965571

[ref66] Maddi S. R. Kobasa S. C. (1984). The Hardy Executive: Health Under Stress. Homewood. IL: Dow Jones-Irwin.

[ref67] Malecki C. K. Demary M. K. (2002). Measuring perceived social support: development of the child and adolescent social support scale (CASSS). Psychol. Sch. 39, 1–18. doi: 10.1002/pits.10004

[ref68] Malkin V. Rogaleva L. Kim A. Khon N. (2019). The hardiness of adolescents in various social groups. Front. Psychol. 10:2427. doi: 10.3389/fpsyg.2019.02427, PMID: 31708851 PMC6824409

[ref69] McDonald R. P. Ho M. H. R. (2002). Principles and practice in reporting structural equation analyses. Psychol. Methods 7, 64–82. doi: 10.1037/1082-989X.7.1.64, PMID: 11928891

[ref71] Ministry of Education of the PRC and Ministry of Finance of the PRC, ed. (2007). No. 8 Document in 2007-Guidance from the ministry of education and finance on identifying students with financial difficulties in institutions of higher education. Available at: http://www.moe.gov.cn/jyb_xxgk/gk_gbgg/moe_0/moe_1443/moe_1581/tnull_25283.html (Accessed March 19, 2022).

[ref72] Ministry of Education of the PRC, Ministry of Finance of the PRC, Ministry of Civil Affairs of the PRC, Ministry of Human Resources and Social Security of the PRC, State Council Poverty Alleviation Office, and China Disabled Persons Federation. ed. (2018). No. 16 Document in 2018-Guidance from The Ministry of Education and other six relevant ministries on Identifying for Students with Financial Difficulties. http://www.moe.gov.cn/srcsite/A05/s7505/201811/t20181106_353764.html (Accessed March 10, 2022).

[ref73] Moscovitch D. A. Hofmann S. G. Litz B. T. (2005). The impact of self-construals on social anxiety: a gender-specific interaction. Pers. Individ. Differ. 38, 659–672. doi: 10.1016/j.paid.2004.05.021.2005

[ref74] Muda I. Ambarita H. Lukman I. B. Siahaan A. Y. (2016). Hardiness of Karo survivors affected by Sinabung eruption based on gender. Adv. Soc. Sci. Educ. Hum. Res. 81, 225–229. doi: 10.2991/icosop-16.2017.33

[ref75] Murphy R. (2022). What does ‘left behind’ mean to children living in migratory regions in rural China? Geoforum 129, 181–190. doi: 10.1016/j.geoforum.2022.01.012

[ref76] Neissi A. Shehni Yeylagh M. Frashbandi A. (2005). A study of simple and multiple relationships of self. Esteem, general anxiety, perceived social support and psychological hardiness with social anxiety in first grade female high school students in Abadan. J. Psychol. Achiev. 12, 137–152. doi: 10.22055/PSY.2005.16358

[ref77] Nezhad M. A. S. Besharat M. A. (2010). Relations of resilience and hardiness with sport achievement and mental health in a sample of athletes. Procedia Soc. Behav. Sci. 5, 757–763. doi: 10.1016/j.sbspro.2010.07.180/

[ref78] Nunnally J. C. Bernstein I. (1994). Psychometric Theory (3rd Edn.). New York, NY: McGraw-Hill.

[ref79] Pan M. Zhang S. Q. Zhou S. S. Cong T. K. Tao M. Y. Han Y. D.. (2021). Analysis of related factors and coping styles of college students’ the mental health under stress. Chin. J. Health Psychol. 29, 309–313. doi: 10.13342/j.cnki.cjhp.2021.02.032

[ref80] Pengilly J. W. Dowd E. T. (2000). Hardiness and social support as moderators of stress. J. Clin. Psychol. 56, 813–820. doi: 10.1002/(SICI)1097-4679(200006)56:6<813::AID-JCLP10>3.0.CO;2-Q, PMID: 10877469

[ref81] Podsakoff P. M. MacKenzie S. B. Lee J. Y. Podsakoff N. P. (2003). Common method biases in behavioral research: a critical review of the literature and recommended remedies. J. Appl. Psychol. 88, 879–903. doi: 10.1037/0021-9010.88.5.879, PMID: 14516251

[ref82] Purdon C. Antony M. Monteiro S. Swinson R. P. (2001). Social anxiety in college students. J. Anxiety Disord. 15, 203–215. doi: 10.1016/S0887-6185(01)00059-7 11442139

[ref83] Qiu K. G. Dong B. Cui Y. C. (2011). Research on anxiety and its influencing factors in poor college students. Chin. J. Health Psychol. 19, 1378–1379. doi: 10.13342/j.cnki.cjhp.2011.11.027

[ref84] Rapee R. M. Fardouly J. Forbes M. K. Johnco C. Magson N. R. Oar E. L.. (2019). Adolescent development and risk for the onset of social-emotional disorders: a review and conceptual model. Behav. Res. Ther. 123, 103501. doi: 10.1016/j.brat.2019.103501, PMID: 31733812

[ref85] Ren Y. J. Li M. L. (2020). Influence of physical exercise on social anxiety of left-behind children in rural areas in China: the mediator and moderator role of perceived social support. J. Affect. Disord. 266, 223–229. doi: 10.1016/j.jad.2020.01.152, PMID: 32056881

[ref86] Robnett R. D. Anderson K. J. (2017). Feminist identity among women and men from four ethnic groups. Cultur. Divers. Ethnic Minor. Psychol. 23, 134–142. doi: 10.1037/cdp0000095, PMID: 27845525

[ref87] Rubenstein J. C. (2013). Pluralism about global poverty. Br. J. Polit. Sci. 43, 775–797. doi: 10.1017/S0007123412000385

[ref88] Sarason I. G. Levine H. M. Basham R. B. Sarason B. R. (1983). Assessing social support—the social support questionnaire. J. Pers. Soc. Psychol. 44, 127–139. doi: 10.1037/0022-3514.44.1.127

[ref89] Schlenker B. R. (2012). “Self-presentation,” in Handbook of Self and Identity. eds. Leary M. R. Tangney J. P. (New York: Guilford Publications), 542–570.

[ref90] Schlenker B. R. Leary M. R. (1982). Social anxiety and self-presentation: a conceptualization model. Psychol. Bull. 92, 641–669. doi: 10.1037/0033-2909.92.3.641, PMID: 7156261

[ref91] Stoeckli G. (2010). The role of individual and social factors in classroom loneliness. J. Educ. Res. 103, 28–39. doi: 10.1080/00220670903231169

[ref93] Tabachnick B. G. Fidell L. S. (2001). Using Multivariate Statistics. 4th Edn, Allyn and Bacon, Boston.

[ref94] Taheri A. Ahadib H. Kashani F. L. Kermani R. A. (2014). Mental hardiness and social support in life satisfaction of breast cancer patients. Procedia Soc. Behav. Sci. 159, 406–409. doi: 10.1016/j.sbspro.2014.12.397

[ref95] Tan L. Wu X. X. (2017). “Path research on ideological and political education for poverty-stricken college students based on psychological analysis,” in Proceedings Paper of the 7th International Conference on Education and Management (ICEM 2017). Vol. 53. eds. Wang Z. Miracle J. Kun Z. (Atlantis Press), 303–306.

[ref96] Taylor S. E. Sherman D. K. Kim H. S. Jarcho J. Takagi K. Dunagan M. S. (2004). Culture and social support: who seeks it and why? J. Pers. Soc. Psychol. 87, 354–362. doi: 10.1037/0022-3514.87.3.354, PMID: 15382985

[ref97] Thoits P. A. (1995). Stress, coping and social support processes: where are we? What next? J. Health Soc. Behav. 35, 53–79. doi: 10.2307/2626957 7560850

[ref98] Turk C. L. Heimberg R. G. Orsillo S. M. Holt C. S. Gitow A. Street L. L.. (1998). An investigation of gender differences in social phobia. J. Anxiety Disord. 12, 209–223. doi: 10.1016/S0887-6185(98)00010-3, PMID: 9653680

[ref99] Van Zalk N. Van Zalk M. H. W. (2015). The importance of perceived support by fiends and parents for adolescent social anxiety. J. Pers. 83, 346–360. doi: 10.1111/jopy.12108, PMID: 24957362

[ref100] Wang Y. Li Y. P. Li N. Hang R. J. (2015). A survey on mental health status among impoverished students in a college of Guangxi ethnic areas. Chin. J. Health Educ. 31, 467–469+496. doi: 10.16168/j.cnki.issn.1002-9982.2015.05.009

[ref101] Watson D. Friend R. (1969). Measurement of social-evaluation anxiety. J. Consult. Clin. Psychol. 33, 448–457. doi: 10.1037/h0027806, PMID: 5810590

[ref102] Weeks J. W. Heimberg R. G. Rodebaugh T. L. Norton P. J. (2008). Exploring the relationship between fear of positive evaluation and social anxiety. J. Anxiety Disord. 22, 386–400. doi: 10.1016/j.janxdis.2007.04.009, PMID: 17531437

[ref103] Weinstock L. S. (1999). Gender differences in the presentation and management of social anxiety disorder. J. Clin. Psychiatry 60, 9–13. 10335674

[ref104] Weymouth B. B. Buehler C. (2018). Early adolescents’ relationships with parents, teachers, and peers and increases in social anxiety symptoms. J. Fam. 32, 496–506. doi: 10.1037/fam0000396, PMID: 29620376 PMC5991991

[ref105] Wiebe D. J. (1991). Hardiness and stress moderation: a test of proposed mechanisms. J. Pers. Soc. Psychol. 60, 89–99. doi: 10.1037/0022-3514.60.1.89, PMID: 1995836

[ref108] Yu Y. Liu S. Song M. H. Fan H. Zhang L. (2019). Effect of parent-child attachment on college students’ social anxiety: a moderated mediation model. Psychol. Rep. 123, 2196–2214. doi: 10.1177/0033294119862981 31333073

[ref109] Zeng J. H. Lu A. T. Guo Y. Y. Cai R. Y. (2017). The relationship between social support and social anxiety in college students with financial difficulties: the mediating effect of resilience. Psychol. Res. 10, 83–89.

[ref110] Zhang L. J. (2000). Study on anxiety level and social support of impoverished college students. Chin. Ment. Health J. 14:196.

[ref111] Zhao X. Zhang P. Chen L. Zhou R. L. (2014). Gender differences in the relationship between attentional bias to threat and social anxiety in adolescents. Pers. Individ. Differ. 71, 108–112. doi: 10.1016/j.paid.2014.07.023

[ref112] Zhou H. L. Jiang H. B. Zhang B. Liang H. Y. (2021). Social anxiety, maladaptive cognition, mobile phone addiction, and perceived social support: a moderated mediation model. J. Psychol. Africa 31, 248–253. doi: 10.1080/14330237.2021.1927354

[ref114] Zimet G. D. Dahlem N. W. Zimet S. G. Farley G. K. (1988). The multidimensional scale of perceived social support. J. Pers. Assess. 52, 30–41. doi: 10.1207/s15327752jpa5201_2 2280326

